# Exploring the Potential of *Lemon* Peel Extracts in Cosmetics: Chemical Composition and Bioactive Properties

**DOI:** 10.4014/jmb.2412.12042

**Published:** 2025-03-26

**Authors:** Jinqin Huang, Xu Xu, Chang Liu, Liping Liu

**Affiliations:** 1 Department of Biological Sciences, College of Biological and Environmental Sciences, Zhejiang Wanli University, Ningbo 315100, P.R. China; 2 Research and Development Department, Ningbo Dayang Technology Co. Ltd., Ningbo 315031, P.R. China

**Keywords:** *Lemon* peel extracts, ingredient, aroma, antimicrobial, bioactivity, cosmetic raw material

## Abstract

In this study, the potential of *lemon* peel extracts as cosmetic raw materials was explored, with focus on their composition, aroma, antimicrobial, and other bioactivities. Lemon peel essential oil (LPEO), extract (LPE) and absolute oil (LPAO) were prepared by hydrodistillation and organic solvent extraction, respectively. GC/MS analysis indicated that LPEO, LPE, and LPAO contained 22, 39, and 9 components, respectively, with terpenoids being the predominant component. LPE had the highest total flavonoid content and exceeded the total phenolic content. LPEO demonstrated the strongest aroma intensity and persistence, as measured by electronic nose. All three *lemon* peel extracts showed good antioxidant, anti-tyrosinase, and antimicrobial properties, while inhibition rates exceeded 90% in the experimental concentration range in a dose-dependent manner. Although the antioxidant and antibacterial effects of LPAO were stronger than those of LPEO, the latter had better anti-tyrosinase action. LPEO also demonstrated superior anti-inflammatory effects, with inhibitory rates of 87.79 ± 3.86% and 80.75 ± 2.33% on TNF-α and IL-6 at 1 × 10^-2^ mg/ml. Moreover, LPEO promoted HaCaT cell migration better than LPE and LPAO, and the healing rate of scratched HaCaT cells treated with LPEO at 1 × 10^-2^ mg/ml for 12 h was 95.29 ± 3.41%. In addition to its antioxidant and antibacterial properties, the overall performance of LPEO was superior by comparison with LPE and LPAO. In summary, the three extracts can be combined to expand their application as skincare and cosmetic additives with aroma-improving, antioxidant, whitening, antibacterial, anti-inflammatory, and wound-healing activities.

## Introduction

Current trends in cosmetic formulations are developing towards a more natural and chemical-free approach [1, 2]. Along with active ingredients, cosmetic products often include artificial flavors to enhance fragrance, and preservatives to extend the shelf life. However, synthetic fragrances generally lack biological activity, and some can also cause skin allergies. Meanwhile, preservatives such as methylchloroisothiazolinone/methylisothiazlinone and formaldehyde-releasing agents can result in contact dermatitis [3]. The overuse of these harmful substances can even lead to the emergence of drug-resistant strains, posing a serious risk to consumers [4].

The essential oil extracted from natural plants has a series of biological activities, including aroma, antioxidant, anti-aging, antibacterial, anti-acne and anti-inflammatory properties [5]. *Lemon*, as the third largest variety of citrus in Rutaceae, is famous for its dual application in both medicine and food. *Lemon* peel accounts for about 20% of the whole *lemon*, which is rich in terpenes, alkaloids, phenolic acids and other active compounds, and has a pleasant aroma and unique biological activities [6]. Scientific research has verified the benefits of *lemon* essential oil in promoting microvascular circulation, promoting subcutaneous fat combustion, stimulating collagen synthesis, restoring the skin’s oil-water balance, and inhibiting melanin production [7]. Furthermore, *lemon* essential oil could modulate cell permeability and demonstrate potent inhibitory effects against *Aeromonas aeruginosa*. The terpenoids in *lemon* peel extract had strong antioxidant activity and had obvious scavenging effect on DPPH and ABTS free radicals [8]. The (*Z*)-citral found in *lemon* extract demonstrated a potent inhibitory effect on tyrosinase, thereby promoting skin whitening [9]. Furthermore, (*Z*)-citral has been shown to alleviate LPS-induced cell damage by inhibiting inflammation, oxidative stress, and epithelial-mesenchymal transition in THLE-2 cells. It might also influence the brain via the olfactory system, potentially regulating anxiety and depression [10]. *Lemon* peel is rich in antifungal compounds such as limonene, *β*-pinene, and γ-terpene, which could disrupt the balance of organelles and inhibit the metabolism of *C. albicans* [11]. Neryl acetate and limonene in *lemon* essential oil are the key components of advanced aromatic agents. The flavonoids and polyphenols contained in *lemon* peel extract had effectively scavenged free radicals, inhibited tyrosinase activity, and reduced the production of NO by macrophage RAW264.7 cells [12], while also exhibiting strong antibacterial effects against *Staphylococcus aureus* and *Escherichia coli* [13].

The current research on *lemon* peel mainly focuses on the extraction and application of *lemon* essential oil, highlighting its antioxidant and antibacterial properties, and its application in the field of food industry. However, with the growing preference for natural ingredients in the cosmetics industry, there is a high demand for plant extracts with natural aroma, antibacterial properties, and biological activities. In this paper, the *lemon* peel extracts obtained by three different extraction methods were compared from the aspects of composition, aroma, antioxidant, anti-inflammatory, antibacterial, whitening potential, cytotoxicity, and cell repair ability. The aim is to develop a *lemon* peel extract with enhanced flavor and antibacterial and antiseptic properties, as well as a variety of biological activities, so as to provide the basis for its application as a cosmetic raw material.

## Materials and Methods

### Reagents

Rutin, gallic acid, arbutin, ascorbic acid, folin phenol, polyphenol oxidase, L-tyrosine, aluminum chloride hexahydrate, potassium persulfate, sodium nitrite, phosphate - buffered saline (PBS, pH6.8), 2,2’-azino-bis (3-ethylbenzothiazoline- 6-sulfonic acid) diammonium salt (ABTS), 1,1-diphenyl-2-picrylhydrazide (DPPH), as well as solvents (ethanol, dimethylsulfoxide (DMSO), petroleum ether, propylene glycol, sodium chloride) were analytical grade and purchased from Sinopharm (China). HaCaT cell lines (No.KMCC-001-0256), Luria-Bertani medium (LB medium), recombinant broth, fetal bovine serum (FBS), cell counting kit-8 (CCK-8), tumor necrosis factor-α (TNF-α) and interleukin-6 (IL-6) ELISA kits were purchased from Beina Biological Co. (China). *E. coli* (No. CMCC(B)44102), *S. aureus* (No. CMCC(B)26003) and *Candida albicans* (*C. albicans*, No. CMCC(B)98001) were gifted by Zhang Jie (Zhejiang Wanli University). *Lemon* was purchased in Anyue, Sichuan, China and identified as *Citrus* × *Limon* '*Eureka*' by Professor Liu Liping.

### Instruments and Equipment

Electronic balance (BSA124S, Sartorius, Germany); rotary evaporator (RV10, Ika, Country); gas chromatography-mass spectrometry (8890-5977B, Agilent, Country); electronic nose (PEN3.5, Airsense, Country); spectrophotometer (TU-1810, Beijing general instruments, Country); high-speed centrifuge (5417R, Sigma); microplate reader (FC, Thermo Fisher Scientific, Country); incubator (THZ-D, Ningbo Southeast Instruments); carbon dioxide incubator (4111FO, Thermo Fisher Scientific).

### Preparation of *Lemon* Peel Extracts

The *lemon* peel was first washed and then dried at 40°C for 2~4 h before being pulverized and passed through a 10-mesh sieve for later use.

*Lemon* peel essential oil (LPEO) was extracted using the steam distillation method. *Lemon* peel and distilled water (with 2% w/v NaCl) at a solid-liquid ratio of 1:10 were placed in a distillation flask for a micro-boiling reflux extraction process, continuing until the distillate was free of oil droplets. The resulting oily liquid was separated using an oil-water separator, dried with anhydrous Na_2_SO_4_, and stored in a brown bottle.

*Lemon* peel extract (LPE) was prepared using the ethanol extraction method. A total of 100 g of *lemon* peel was extracted twice with 500 ml of 90% v/v ethanol containing 1% v/v propylene glycol at 40°C for durations of 4 h and 2 h, respectively. The supernatants were combined, filtered, and evaporated to obtain the extract, which was then sealed in a brown bottle.

*Lemon* peel absolute oil (LPAO) was obtained using the petroleum ether extraction method. A total of 100 g of *lemon* peel was extracted to two reflux extractions with 500 ml of petroleum ether at 40°C for durations of 4 h and 2 h, respectively. The supernatants from both extractions were combined, filtered, and evaporated into a paste. Anhydrous ethanol was added to the paste at a volume ratio of 10:1, stirred well, and allowed to stand at -20°C for 4 h. The resulting supernatant was filtered, and evaporated, leading to the acquisition of the absolute oil, which was stored in a brown bottle.

### Gas Chromatography-Mass Spectrometry (GC/MS) Detection of Components

All three samples were diluted with n-hexane.

GC conditions: The HP-5MS capillary column used in this study was 30 m × 0.25 mm × 0.25 μm. Helium served as the carrier gas, with a flow rate of 1 ml/min. The injection was performed with a split ratio of 200:1 and an injection sample volume of 1 μl at 220°C. The initial temperature was set at 50°C and maintained for 3 min, then increased by a rate of 2°C/min up to 140°C for 12 min, 10°C/min up to 250°C for 5 min. The total operating time for this procedure was 84 min.

MS conditions: An electron ionization source (EI) was utilized with an electron energy of 70 eV. The temperature of the ion source, the quadrupole, the transmission line was 230°C, 150°C, and 270°C, respectively. The full scanning range covered a mass-to-charge ratio (m/z) of 35~550. The identification of each volatile substance was performed using the National Institute of Standards and Technology (NIST) MS spectrometry library, while the relative content was calculated employing the area normalization method.

### Determination of Total Flavonoid and Phenolic Content

Total flavonoid content was determined in the samples by spectrophotometry using rutin as a control, with reference to source [15]. Five ml of rutin standard solution was prepared with concentrations ranging from 0 to 100 μg/ml using dimethyl sulfoxide (DMSO) as the solvent. To this solution, 0.3 ml of 5% NaNO_2_, 0.3 ml of 10% w/v AlCl_3_·6H_2_O and 4 ml of 4% w/v NaOH were added, and the mixture was allowed to react at room temperature for 15 min. The absorbance at 510 nm (*A*_510nm_) was measured with deionized water as a blank. The standard curve for rutin was described by the equation *A* = 0.0067C + 0.0098 (*r* = 0.9978). Three *lemon* peel extracts were diluted with DMSO and analyzed using the same method.

Total phenolic content was determined using the Folin-Ciocalteu colorimetric method [16]. Standard solutions of gallic acid, ranging from 0 to 120 μg/ml, were prepared in DMSO. Then, 4 ml of distilled water, 0.5 ml of Folin reagent, and 4 ml of 8% w/v Na_2_CO_3_ solution were added to each standard solution. The reaction was conducted at room temperature in the dark for 30 min. The absorbance at 740 nm (*A*_740nm_) was measured with deionized water as a blank. The standard curve for gallic acid was established as *A* = 0.0104C + 0.0021 (*r* = 0.9998). Subsequently, the sample solutions of *lemon* peel extracts were prepared in DMSO and 1 ml of the dilution solution was used to determine the total phenolic content following the same procedure.

### E-Nose Detection of Odor

Odor was detected in headspace aspiration with PEN3 e-nose [17]. The experimental parameters were set as follows: a collection time of 300 s, with a data collection cycle of 1 s, and a collection delay of 120 s. The injection volume was 2.0 ml with the flow rate of 150 ml/min. Each sample underwent an average incubation time of 2 min at a temperature of 35°C, with a stirring speed of 500 r/min. The sensor was programmed for a self-cleaning time of 150 s, followed by a zeroing period of 5 s. Data analysis was conducted using WinMuster software in conjunction with the PEN 3 e-nose.

### Determination of Free Radical Scavenging Rate

The antioxidant activity of *lemon* peel extracts was assessed using the DPPH and ABTS methods as described by Patricia [18]. Half-scavenging concentration (*IC_50_*) refers to the concentration of *lemon* peel extract required to eliminate 50% of DPPH and ABTS free radicals.

### Determination of Anti-Tyrosinase Activity

The experiment involved four groups: Group A, which contained 1.5 ml of 50 U/ml tyrosinase, Group B, which did not contain it, Group C, which contained 1.5 ml of 50 U/ml tyrosinase and 1 ml of sample solution, and Group D, which contained only 1 ml of sample solution. All groups were supplemented with PBS buffer to a total volume of 5 ml. The samples were then incubated for 10 min at 37°C, and immediately 2.5 mmol/l tyrosine solution was added to initiate the reaction in the dark for 30 min at 37°C. The absorbance at 475 nm was determined for each set of samples with deionized water as a blank. Arbutin was used as a positive control. The inhibition rate of tyrosinase was calculated as follows [19]:

*Inhibition rate/%*=[(*A_A_-A_B_*)-(*A_C_-A_D_*)]×100/(*A_A_-A_B_*).

### Determination of Antibacterial Activity

Preparation of bacterial suspension [20]: *E. coli* and *S. aureus* were activated and cultured at 37°C and *C. albicans* at 28°C to logarithmic growth phase. The bacteria were centrifuged and washed twice with 0.9% w/v normal saline, resuspended in LB medium, and the bacterial concentration was adjusted to 1 × 10^8^ ~5×10^8^ CFU/ml.

Preparation of sample solution: The sample was first dissolved in sterile water containing 8% DMSO, and then diluted with LB medium to concentrations of 0.5, 2.5, 5, 12.5, and 25 mg/ml, respectively, and filtered through a 0.22 μm filter for later use.

Bacteria grouping: Experiments were performed in sterile 96-well plates. The control group was 30 μl bacterial suspension and 170 μl LB medium; the blank group was 200 μl LB medium; the sample group was 30 μl bacterial suspension and 170 μl sample solutions; the background group was 30 μl LB medium and 170 μl sample solutions. After incubating for 24 h at 37°C, the absorbance at 600 nm was measured using a microplate reader. The inhibition rate was the following equation:

*Inhibition rate*/%=[(*A_control_-A_blank_*)-(*A_sample_-A_background_*)]×*100*/(*A_control_-A_blank_*).

### Determination of Cytotoxicity and Anti-Inflammatory Properties

The cytotoxicity on HaCaT cells was determined using the CCK-8 method. Logarithmically grown HaCaT cells (5×10^4^ cells/ml) were seeded at 100 μl/well in 96-well plates and incubated in a CO_2_ incubator overnight at 37°C. The three *lemon* peel extracts were dissolved in 0.05% DMSO and subsequently diluted to six concentrations (1×10^-4^ ~ 10 mg/ml) with DMEM medium containing 10% FBS. Adherent cells and 100 μl of medium were added to the control group; only 100 μl of medium was added to the blank group; the sample group included adherent cells and 100 μl of the sample solution; and the background group was supplemented with 100 μl of medium. After a 24 h cell culture period, 10 μl of CCK-8 reagent was added to each well, followed by an additional 4 h incubation. The absorbance of each well was measured at 450 nm using a microplate reader, with six replicates set for each group. The cell survival rate was calculated according to the following formula:

*Cell survival rate/%*=(*A_sample_-A_background_*)×100/(*A_control_-A_blank_*).

The content of inflammatory factors in cells was measured using an ELISA assay kit. Then, 100 μl of 5 × 10^4^ cells/ml logarithmic growth phase HaCaT cells were seeded into a 96-well plate and cultured in an incubator overnight. After discarding the original culture medium, 100 μl of culture medium containing 1 ng/ml TNF-α and 1 ng/ml IFN-γ was added to the model group and sample group to stimulate the cells for 6 h to induce inflammation. The induction medium was then discarded and the cells were washed three times with PBS. Next, 100 μl of culture medium was added to the model group, and 100 μl of culture medium containing samples was added to the sample group. After cultivation in the incubator at 37°C for 24 h, the contents of TNF-α and IL-6 in the supernatant were determined according to the instructions of the ELISA kit, with six replicates set for each group. Meanwhile, the control group was established. The inhibition rate was on the following equation:

*Inhibition rate/%*=(*A_model_-A_sample_*)×100/*A_model_*.

### Repair Experiment on Scratched HaCaT Cells

The samples were prepared using DMEM medium without FBS. Then, 2 ml/well of 3 × 10^5^ cells/ml of HaCaT cells in the logarithmic growth phase were added into a 6-well plate. When the cells were cultured to almost cover the bottom of the well, the cells were scratched by the sterile pipette head and washed multiple times with PBS solution to remove the suspended cells. Following that, 2 ml of DMEM medium containing samples was added into the cells of test groups. Then, 2 ml of DMEM medium without FBS was added into the control group. After the scratch cells were further cultured for 0, 6, 12, and 24 h, the cultures were observed and photos were taken. The scratch area was calculated by ImageJ software. Healing rate/% = (*A*_0_ - *A*_i_) × 100/*A*_0_, where *A*_0_ was the scratch area at 0 h and *A*_i_ was the scratch area at 6, 12 or 24 h.

### Statistical Analysis

The data were expressed as *x ± sd*. GraphPad Prism was used to make all the graphs. One-way analysis of variance (ANOVA) was used to determine the statistical differences among experimental groups, the value of *p* < 0.05 indicated a significant difference.

## Results and Discussion

### Comparison of Sensory Properties of *Lemon* Peel Extracts

Due to different extraction methods, the three extracts had differences in color, state, and aroma, as shown in Table 1.

### GC/MS Results of *Lemon* Peel Extracts

**Fig. 1:**
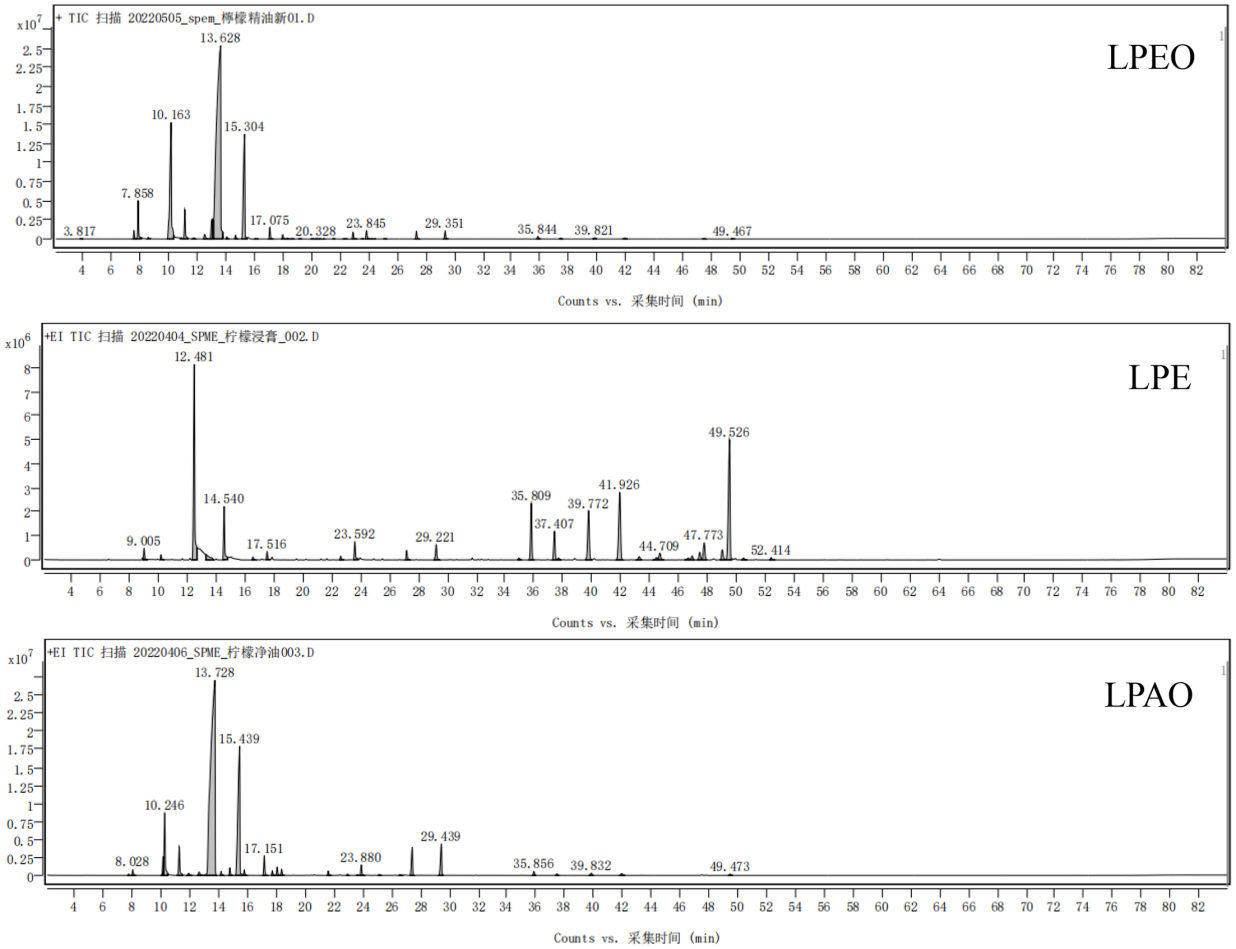
Total ion chromatograms of three *lemon* peel extracts.

According to the total ion chromatograms (Fig. 1) of the three extracts, the detected peaks were shown in Tables 2 and 3.

Chromatographic analysis revealed 22, 39, and 9 distinct peaks in LPEO, LPE and LPAO, respectively. The main components were terpenes, which constituted 81.96% to 96.64% of the extracts, as well as oxygen-containing compounds such as alcohols, aldehydes, and esters. These components are the principal contributors to the aroma of *lemon* peel extracts. Among them, *β*-pinene, myrcene, *D*-limonene, *γ*-terpinene, (*Z*)-citral, linalool and *α*-terpineol were identified in all three extracts. The content of *D*-limonene was the highest in the three extracts, and it has typical orange aroma characteristics [21], antibacterial properties, certain anti-cancer properties [22], as well as effects such as relieving nerve tension and pain, and detoxifying, beautifying and enhancing memory [23]. Terpenoids are the most important aromatic components, with *β*-terpenes serving as precursors for various terpene synthetic fragrances and possessing anti-inflammatory and anti-allergic effects. *γ*-Terpenes had strong antibacterial and antioxidant capabilities, while (*Z*)-citral was noted for its anti-inflammatory, antioxidant and apoptosis inducing properties. Additionally, *α*-terpineol exhibited considerable antioxidant activity [24].

### Total Flavonoid and Phenolic Content in *Lemon* Peel Extracts

The total flavonoid contents in the three *lemon* peel extracts were more than that of total polyphenols, with LPE having the highest content, as shown in Table 4.

Flavonoids and polyphenols possess potent antioxidant properties that effectively eliminate free radicals in the body. These compounds can reduce skin pigmentation caused by reactive free radicals and fatty acids, prevent cellular aging, inhibit the exudation of inflammatory factors [25] and inhibit microbial growth [26].

### E-Nose Results of the Odor of *Lemon* Peel Extracts

**Fig. 2:**
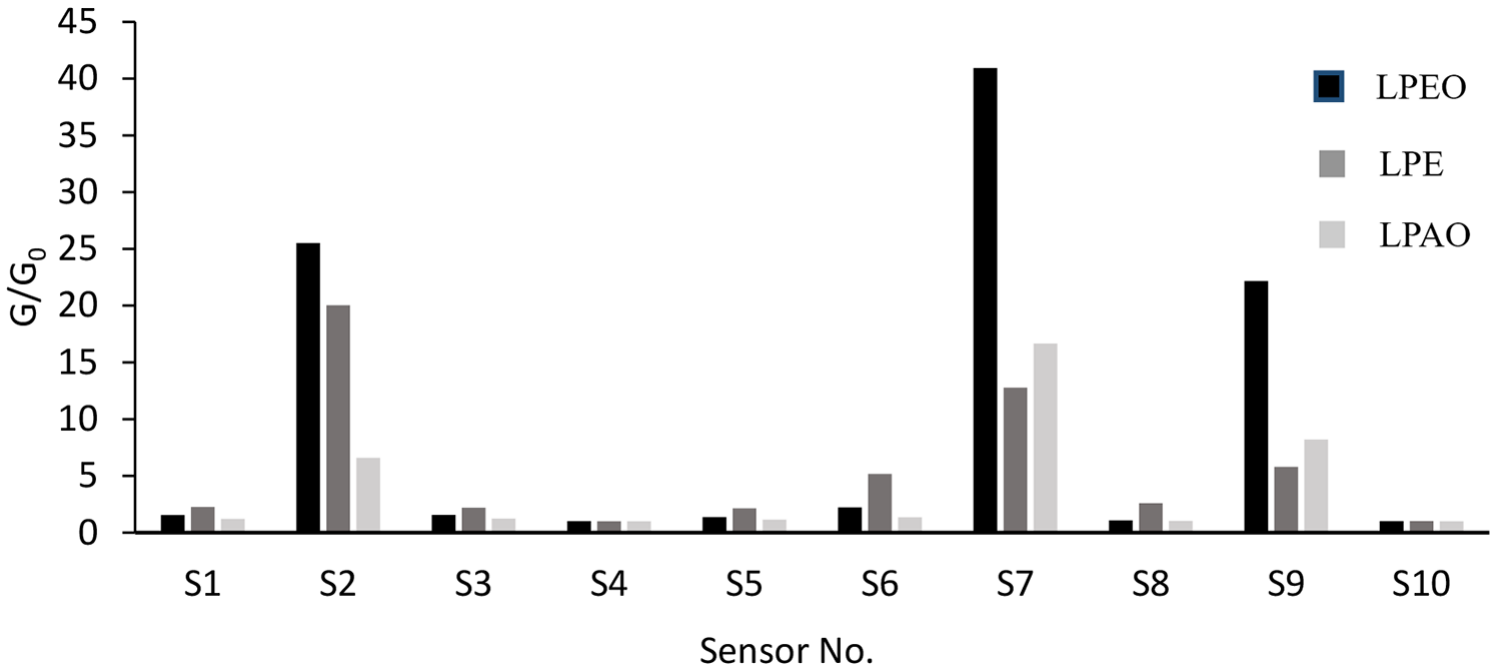
Response values of the odors of three *lemon* peel extracts.

The PEN3 e-nose consists of 10 kinds of sensors. As shown in Fig. 2, the response values were mainly concentrated on the probes of S2 (sensitive to nitrogen oxides), S7 (sensitive to terpenoids and sulfur-containing compounds), and S9 (sensitive to aromatic compounds and organic sulfides). The maximum odor response peaks of three *lemon* peel extracts and the corresponding changes in response values after reaching the peaks of 60, 100, and 140 s were shown in Table 5.

According to the maximum response peak, the intensity of the odor was: LPEO>LPE>LPAO. According to the retention value in peak values within the same time period, the persistence of odor was: LPEO and LPAO > LPE. In addition to terpenoids and aromatic components, LPE also contains some impurities such as plant wax, which affects the odor response values.

### Results of *Lemon* Peel Extracts in Scavenging Free Radicals

**Fig. 3:**
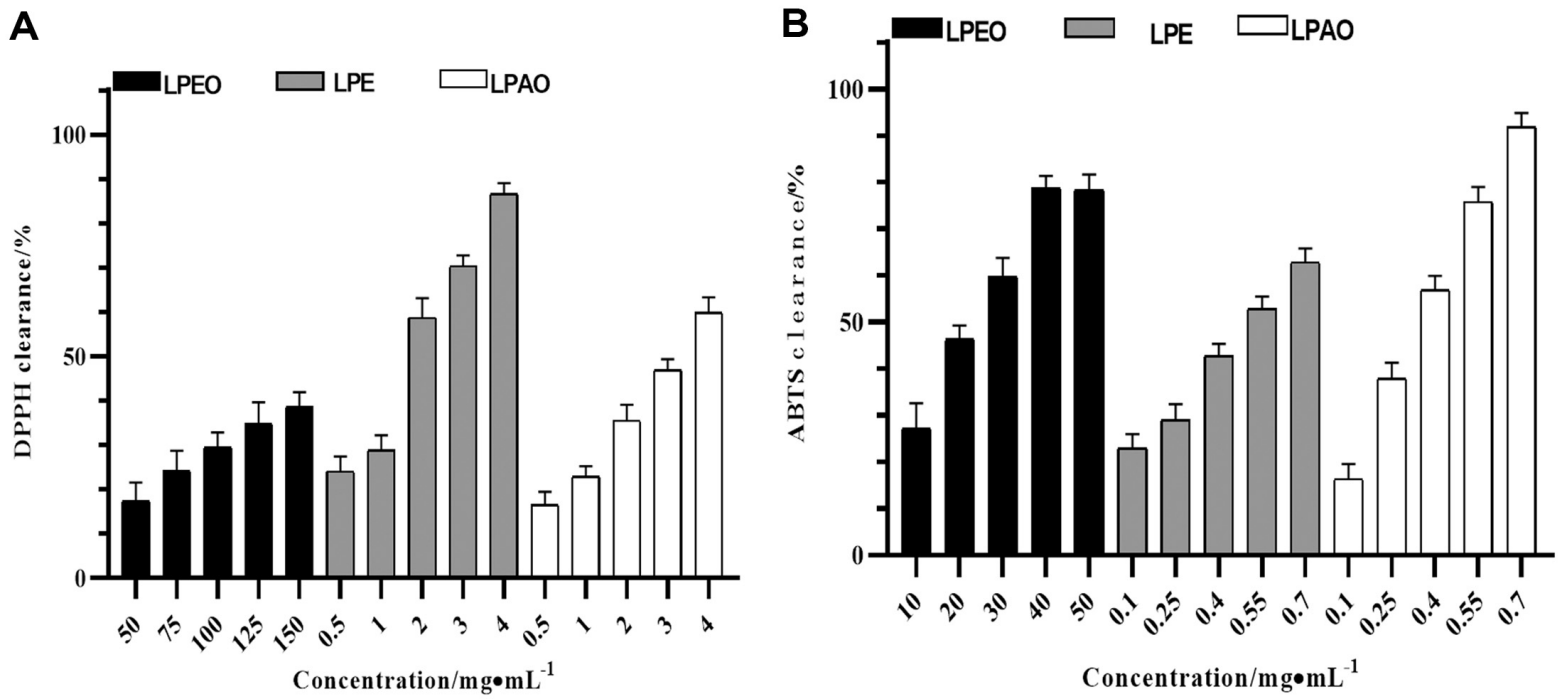
Scavenging rate of DPPH (A) and ABTS (B) by three *lemon* peel extracts (*n* = 3).

The scavenging rate of three *lemon* peel extracts against DPPH and ABTS radicals increased with concentration, as shown in Fig. 3.

The *IC_50_* values of LPEO, LPE, and LPAO were 237.30, 1.53 and 3.15 mg/ml, respectively, and the clearance rate of 4 mg/ml LPE to DPPH was 86.64 ± 1.96%. The *IC_50_* values of LPEO, LPE, and LPAO were 20.76, 0.49 and 0.31 mg/ml, respectively, and the clearance rate of 0.7 mg/ml LPAO to ABTS was 91.80 ± 2.46%. Research found that *D*-limonene had a good antioxidant effect. The contents of (*Z*)-citral, *α*-terpinene and *α*-terpineol were relatively high in LPE, which had strong scavenging effects on DPPH. LPAO contained a higher amount of *γ*-terpinene and (*Z*)-citral, which had strong scavenging effects on ABTS [28].

### Anti-Tyrosinase Activity of *Lemon* Peel Extracts

**Fig. 4:**
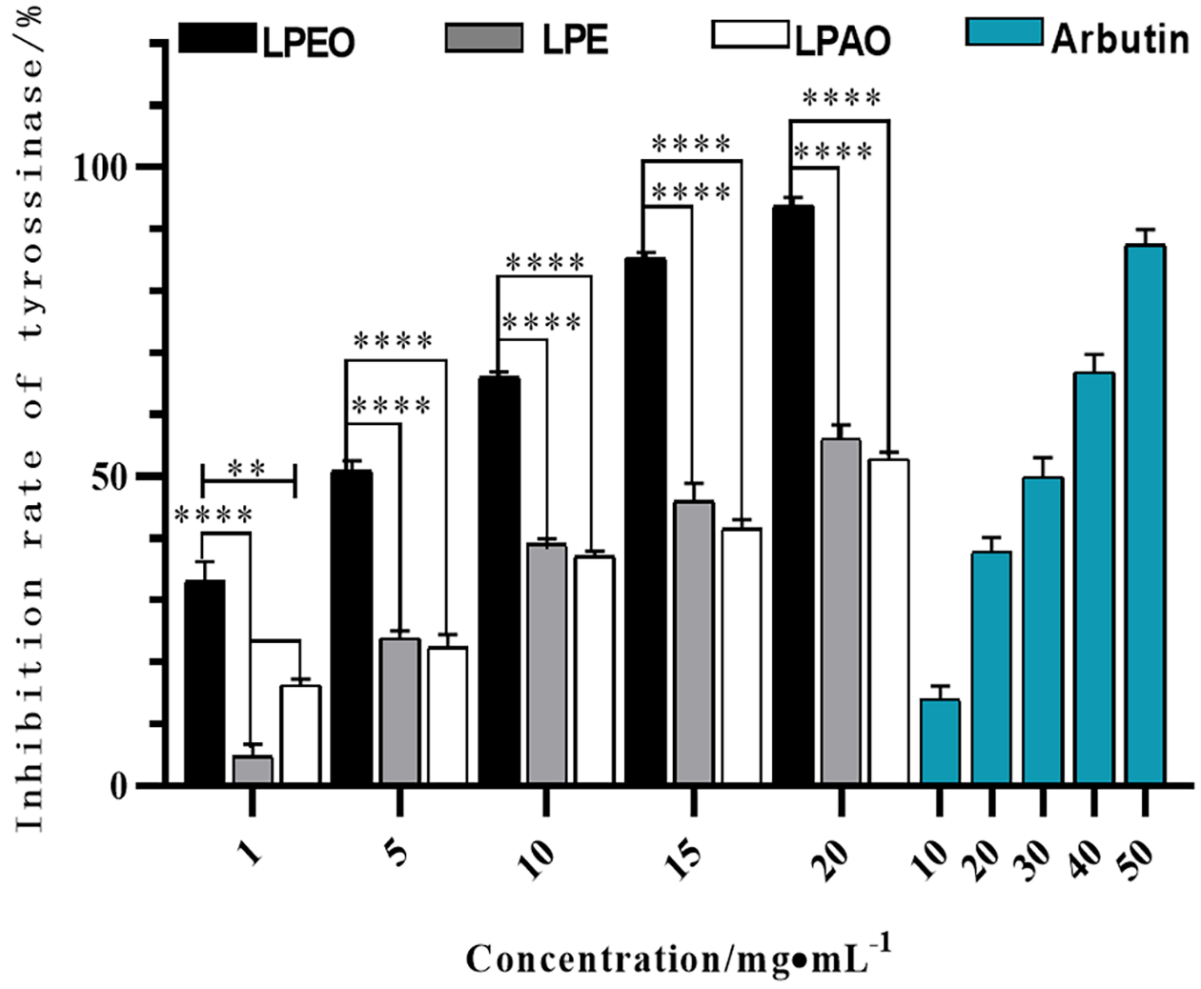
Inhibition effect of *lemon* peel extract on tyrosinase (*n* = 3). **p ≤ 0.01, ***p ≤ 0.001,****p ≤ 0.0001

The inhibitory effect of three *lemon* peel extracts on tyrosinase increased with concentration, as shown in Fig. 4. The inhibition of tyrosinase from the three extracts was LPEO ( *IC_50_* 3.16 mg/ml) > LPE (*IC_50_* 16.02 mg/ml) > LPAO (*IC_50_* 21.73 mg/ml), and the *IC_50_* values of the three extracts were lower than those of the positive control arbutin (26.62 mg/ml). This indicated that their anti-tyrosinase activities were superior to arbutin. The inhibition rate of tyrosinase by LPEO at a concentration of 20 mg/ml was 93.82 ± 1.06%. Studies have shown that the bioactive substances such as the flavonoids and polyphenols rich in *lemon* peel had a similar structure to the substrate of tyrosinase, and acted as substrate analogues to bind with tyrosinase, thereby inhibiting the production of melanin [29]. Meanwhile, the compounds such as (*Z*)-citral, neryl acetate, and *β*-pinene had a significant impact on the activity of tyrosinase [30].

### Antibacterial Activity of *Lemon* Peel Extracts

**Fig. 5:**
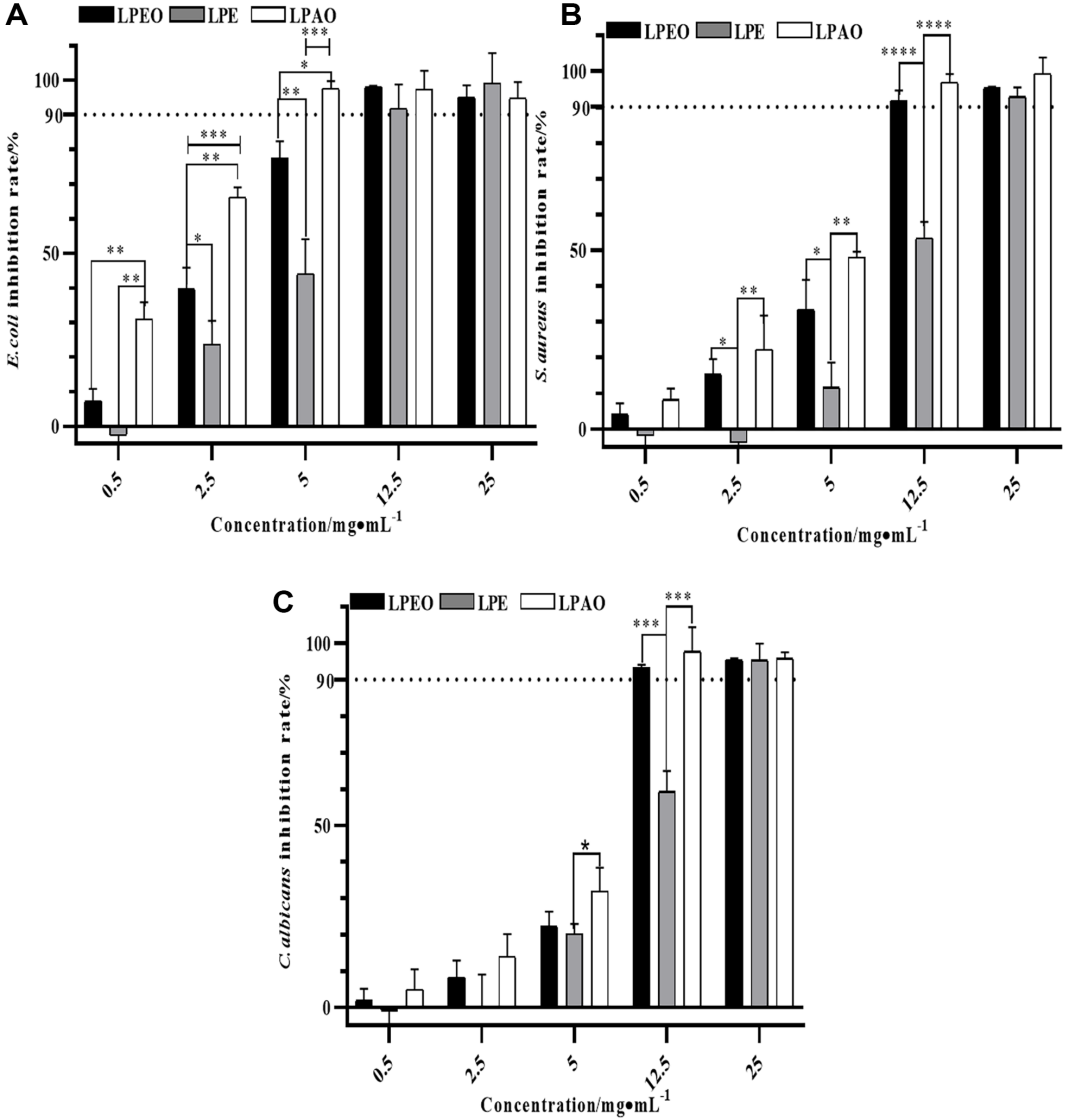
Antibacterial activity of three *lemon* peel extracts against *E. coli* (A) *S. aureus* (B) and *C. albicans* (C) (*n* = 6). *p ≤ 0.05, **p ≤ 0.01, ***p ≤ 0.001, ****p ≤ 0.0001

The antibacterial ability of three *lemon* peel extracts increased with increasing concentration, as shown in Fig. 5. The inhibitory effect of three *lemon* peel extracts on *E. coli* was more sensitive than that on *S. aureus* and *C. albicans*. The minimum inhibitory concentration (MIC) of the three extracts against *E. coli* was as follows: LPAO (4.38 mg/ml) > LPEO (5.79 mg/ml) > LPE (11.89 mg/ml). When the concentrations of LPEO, LPE, and LPAO were 12.53, 23.33, and 11.78 mg/ml, respectively, the antibacterial rates of the three extracts against the three microorganisms exceeded 90%. The antibacterial activity of essential oils is closely related to their chemical composition. Studies have shown that the content of *D*-limonene in *pomelo* peel essential oil was positively correlated with its antibacterial activity (*r* = 0.811~0.923) [31]. Turpentine could damage the biofilms of *S. aureus* and E .coli, leading to increased membrane permeability and inhibited microbial growth. Neryl acetate could damage the ultrastructure of fungi, disrupting their membrane regulatory function and deformability. The content of these compounds in *lemon* peel extracts showed a change of “LPAO > LPEO > LPE” in the GC/MS analysis.

### Cytotoxicity and Anti-inflammatory Effect of *Lemon* Peel Extracts

**Fig. 6:**
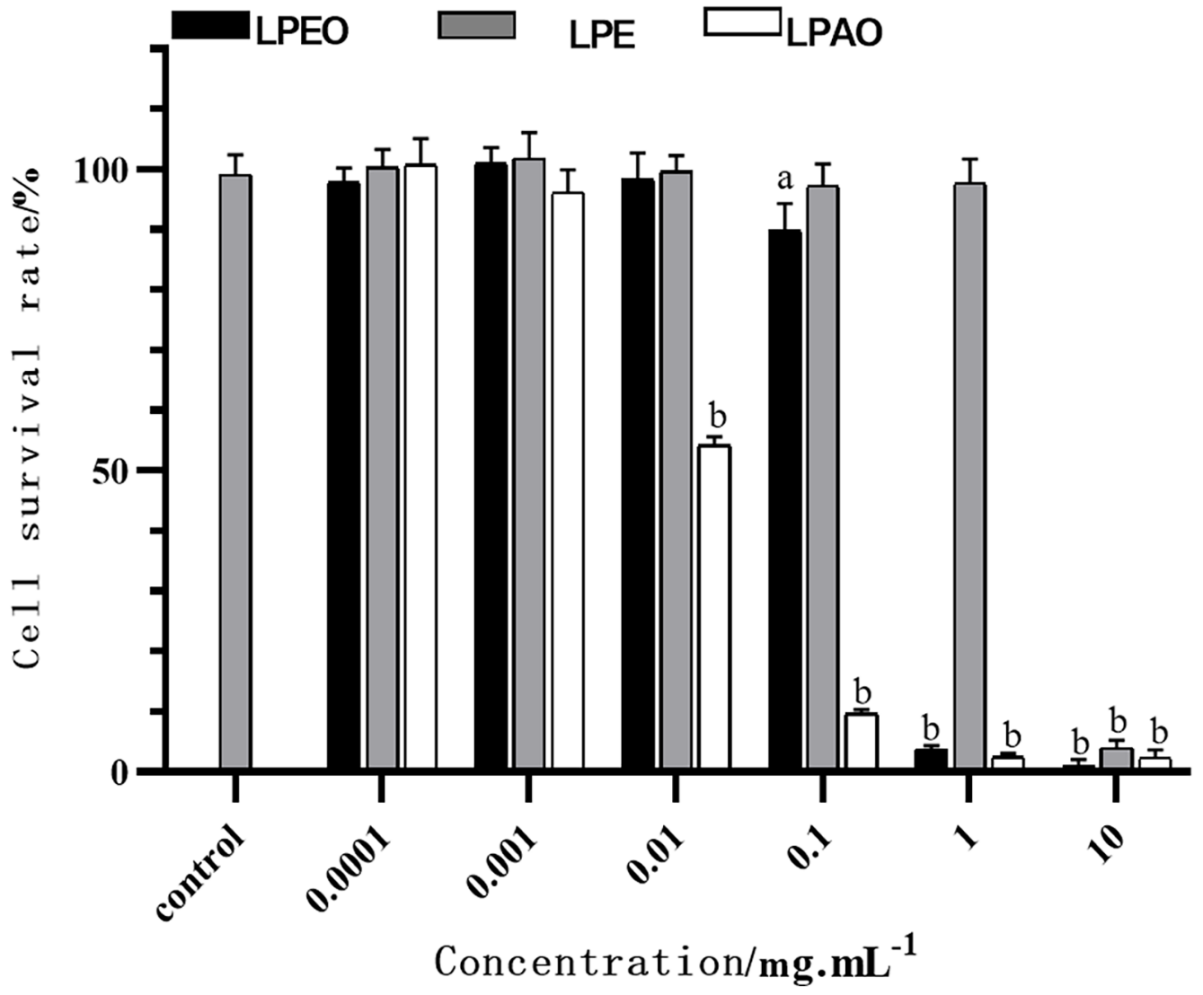
Comparison of toxicity test results of three *lemon* peel extracts on HaCaT cells (*n* = 6). Comparing the sample with the control, a represents *p* ≤ 0.05, and b represents *p* ≤ 0.0001.

The toxicity result of *lemon* peel extracts on HaCaT cells was shown in Fig. 6.

According to Fig. 6, it can be seen that when the concentrations of LPEO, LPE, and LPAO were below 0.01 mg/ml, 1 mg/ml, and 0.001 mg/ml, respectively, the activity of HaCaT cells was greater than 90%. That was to say, the safety sequence of the three extracts for HaCaT cells was: LPE > LPEO > LPAO.

**Fig. 7:**
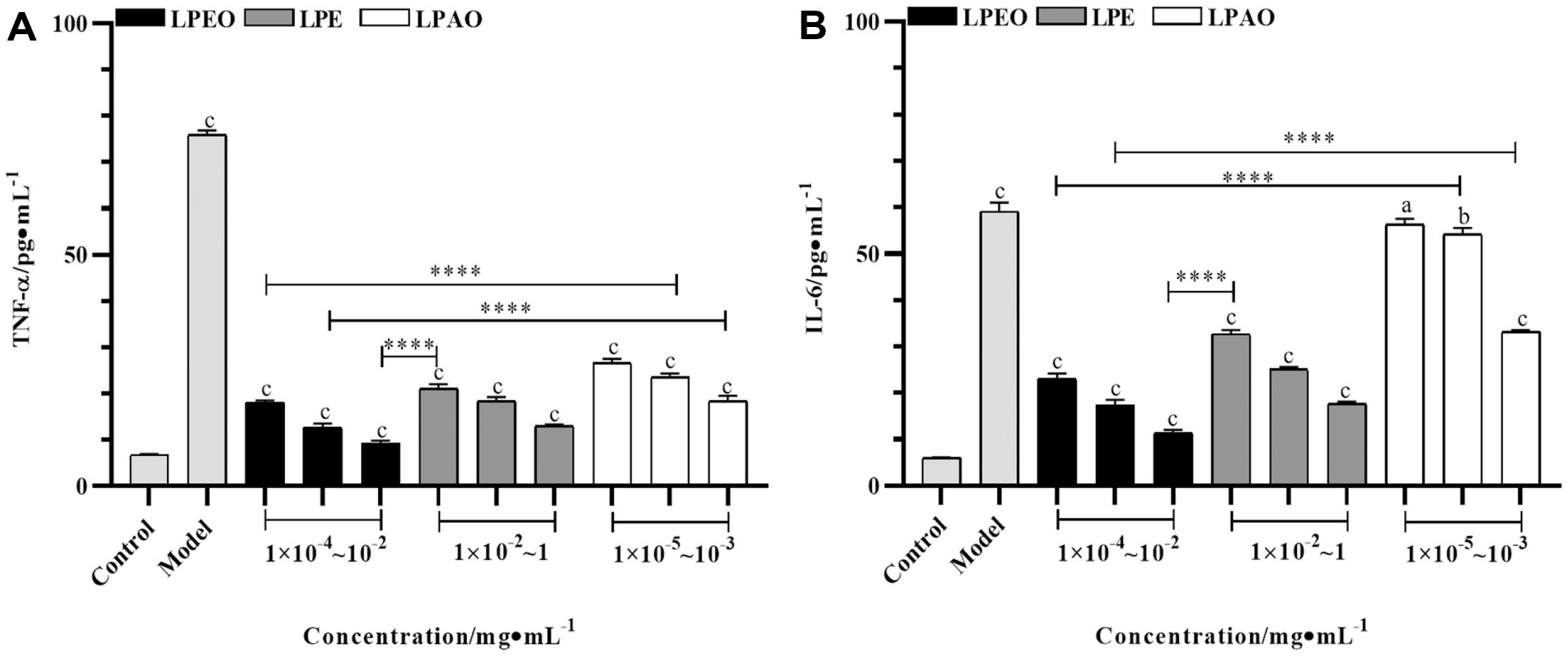
Effects of three *lemon* peel extracts on contents of intracellular TNF-α (A) and IL-6 (B) (*n* = 6). Letters represent the comparison between model group and control group, between sample group and model group. The asterisks represent the comparison between different sample groups of the same concentration. a and * *p* ≤ 0.05, b and ** *p* ≤ 0.01, c and *** *p* ≤ 0.001, **** *p* ≤ 0.001

Subsequent experiments will be conducted within the safe concentration range mentioned above. The results of anti-inflammation (TNF-α, IL-6) were shown in Fig. 7.

There was a significant difference between the model group and the blank group, indicating successful induction of cellular inflammation. Compared with the model group, all three *lemon* peel extracts could effectively inhibit the production of TNF-α and IL-6, and the inhibitory rate increased with dose, but the inflammatory level was not reduced to the cellular state of the blank group. LPEO had the strongest anti-inflammatory effect among the three extracts. The inhibition rates of 1 × 10^-2^ mg/ml LPEO for TNF-α and IL-6 were 87.79 ± 3.86% and 80.75 ± 2.33%, respectively.

The anti-inflammatory properties of essential oils are related to their cascade reactions to cytokines and regulatory transcription factors during signal transduction [32]. Research has confirmed that components such as *D*-limonene, carene, and undecanal had anti-inflammatory effects. For example, *D*-limonene effectively reduced the over expression of inflammatory factors induced by doxorubicin by restoring antioxidant enzyme levels and alleviating oxidative stress [33]. D-lemonene, *δ*-3-carene and *β*-pinene in the essential oil of Chinese torreya effectively inhibit the generation of pro-inflammatory factors and achieve anti-inflammatory effects [34]. Thymol in thyme essential oil had significant inhibitory effects on aging-induced brain inflammation and blood telomere wear in mice [35]. The experimental results were consistent with the GC/MS detection data trends of these substances in three *lemon* peel extracts.

### Repair Results on Scratched HaCat Cells

**Fig. 8:**
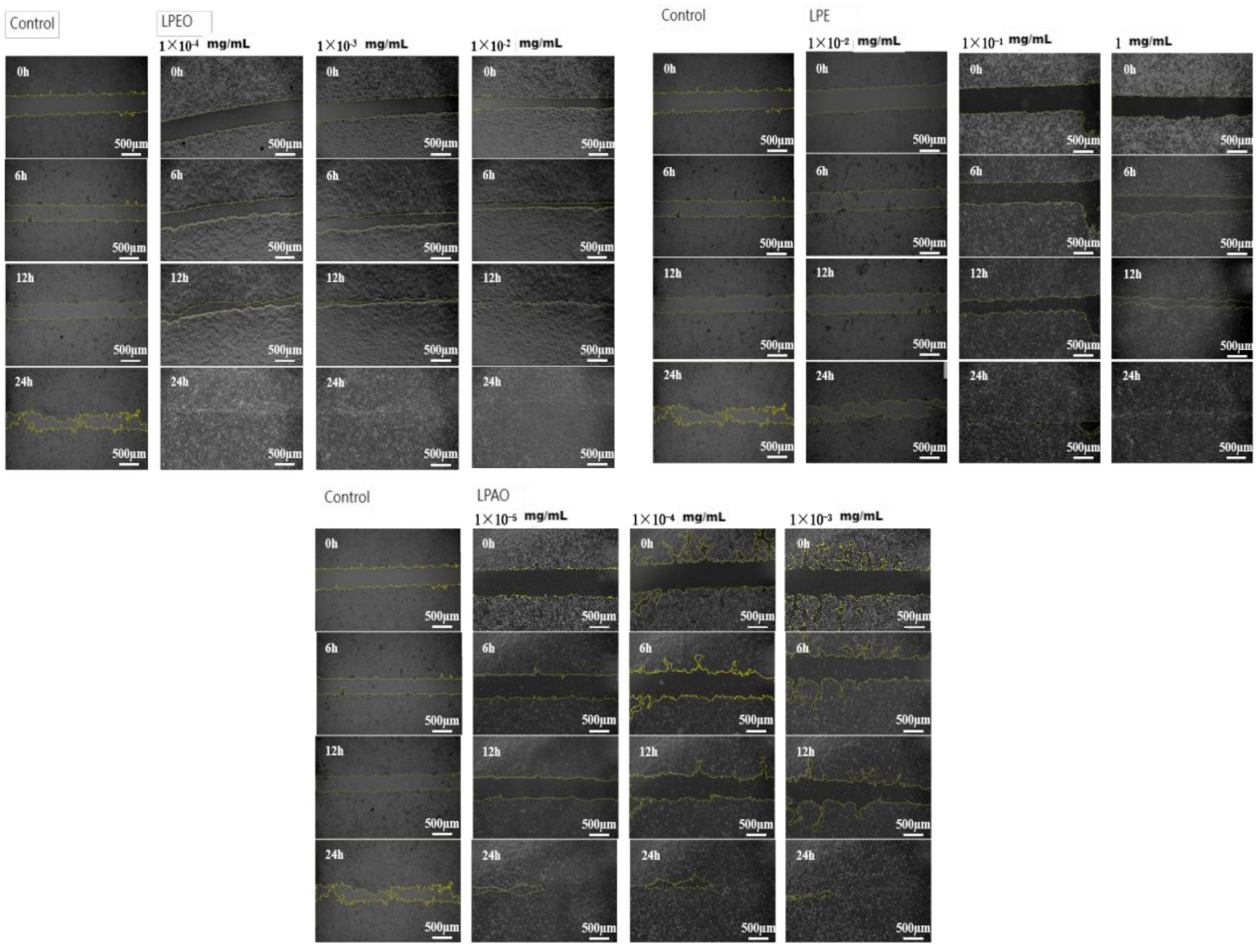
Imaging of repair of scratched HaCaT cells using LPEO, LPE, and LPAO.

**Fig. 9:**
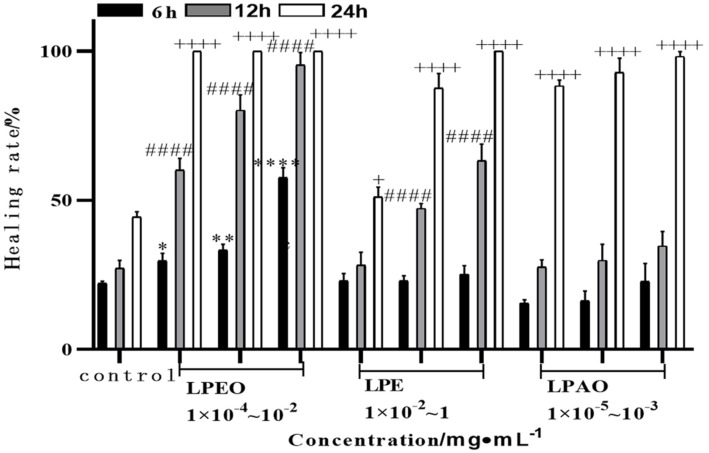
Effects of LPEO, LPE, and LPAO on the healing rate of scratched HaCaT cells. Compared with control group, 6 h: *p <0.05,** *p* <0.01, *** *p* <0.001; 12 h: #*p* <0.05, ## *p* <0.01, ### *p* <0.001; 24 h: +*p* <0.05, ++ *p* <0.01, +++*p* <0.001.

The cell scratch test is commonly used to simulate wound healing *in vitro* [36], and the results were shown in Figs. 8 and 9.

The HaCaT cell healing rate was significantly higher than that of the control group after 24 h of treatment with *lemon* peel extract. With the increase of *lemon* peel extract concentration and the extension of action time, the overall cell healing rate showed an upward trend. Among them, LPEO had a better healing effect on cells than LPE and LPAO. After treating scratched cells with 0.01 mg/ml LPEO for 12 h, the cell healing rate was 95.29 ± 3.41%. Wound repair involves processes such as coagulation, inflammation, cell proliferation, and tissue remodeling. The migration of fibroblasts plays a crucial role in wound healing. Lavender essential oil could promote the transfer of growth factors-*β* and fibroblast growth factors to accelerate wound healing [37]. *Eucalyptus globulus* Labill essential oil also had a good wound-healing effect on rat wound models [38].

### Conclusion

There were differences in the composition of three lemon peel extracts, namely LPE (39 kinds) > LPEO (22 kinds) > LPAO (9 kinds), with the main components being terpenes. The content of total flavonoids in these extracts was higher than that of total polyphenols, and the content of flavonoids in LPE was higher than that in LPAO and LPEO.

The differences in the composition of the three extracts led to the differences in their aroma and biological functions. The aroma intensity and persistence of LPEO were the best among the three extracts.

Among the three *lemon* peel extracts, LPE and LPAO showed superior scavenging abilities against DPPH and ABTS compared to LPEO. LPEO showed a stronger inhibitory effect on tyrosinase than LPE and LPAO, with an inhibition rate of 93.82 ± 1.06% at a concentration of 20 mg/ml, which was higher than that of arbutin. The inhibitory effect of three *lemon* peel extracts on *E. coli* was more sensitive than that on *S. aureus* and *C. albicans*. Among them, compared with MIC values, LPAO had a stronger inhibitory rate on *E. coli* than LPEO and LPE. The safety sequence of the three extracts for HaCaT cells was: LPE (1 mg/ml)> LPEO (0.01 mg/ml) > LPAO (0.001 mg/ml). LPEO showed a stronger inhibitory effect on inflammatory factors compared to LPAO and LPE, with inhibitory rates of 87.79 ± 3.86% for TNF-α and 80.75 ± 2.33% for IL-6 at 1 × 10^-2^ mg/ml. Additionally, LPEO enhanced the migration and healing of HaCaT cells more effectively than LPE and LPAO. Following a 12 h treatment with 1×10-2 mg/ml LPEO, the healing rate of scratched cells reached 95.29 ± 3.41%.

In summary, each of the three extracts has its own characteristics. Overall, LPEO demonstrated superior performance compared to LPE and LPAO. The three extracts can also be combined together to expand their application as cosmetic additives, providing benefits such as enhancing aroma, antioxidant, whitening, antibacterial, anti-inflammatory, and skin wound-healing effects.

## Figures and Tables

**Table 1:** Sensory comparison of three types of *lemon* peel extracts.

Index	LPEO	LPE	LPAO
Color	Colorless transparent	Yellow-brown	Yellow-bright
State	Liquid	Semi-paste	Liquid
Fragrance	Pure and fragrant aroma	Aroma plump	Aroma clear and lasting
Extraction rate/%	4.67 ± 0.28	31.54 ± 1.85	20.42 ± 1.03

**Table 2:** Chemical composition and relative contents of the three *lemon* peel extracts.

No.	Compound	Molecular formula	CAS	Relative percentage content/%		
				LPEO	LPE	LPAO
1	*m*-Cymene	C_10_H_14_	535-77-3	2.93	0.14	-
2	*β*-Phellandrene	C_10_H_16_	555-10-2	0.67	0.16	-
3	*β*-Pinene	C_10_H_16_	18172-67-3	14.28	1.16	5.13
4	Myrcene	C_10_H_16_	123-35-3	2.31	0.43	2.47
5	(+)-Camphene	C_10_H_16_	79-92-5	0.1	-	-
6	*D*-Limonene	C_10_H_16_	5989-27-5	58.05	22.95	63.79
7	(*R*)-Isocarvestrene	C_10_H_16_	1461-27-4	2.20	1.87	-
8	*γ*-Terpinene	C_10_H_16_	99-85-4	11.98	5.74	18.82
9	Terpinolene	C_10_H_16_	586-62-9	0.87	-	2.8
10	Carene	C_10_H_16_	13466-78-9	2.91	0.29	-
11	Tetradecane	C_14_H_30_	629-59-4	-	0.19	-
12	*trans*-Caryophyllene	C_15_H_24_	87-44-5	0.14	7.76	-
13	Santalene	C_15_H_24_	512-61-8	-	0.23	-
14	*α*-Limonene	C_15_H_24_	17699-05-7	0.12	11.82	-
15	1, 5, 9, 9-tetramethyl-1, 4, 7-cyclodecatriene	C_15_H_24_	1000062-61-9	-	0.57	-
16	*β*-Sandalene	C_15_H_24_	25532-78-9	-	0.48	-
17	*β*-Fucoidin	C_15_H_24_	28973-97-9	-	1.15	-
18	*α*-Terpinene	C_15_H_24_	451-55-8	-	0.4	-
19	*β*-Sesquiphellandrene	C_15_H_24_	20307-83-9	-	0.62	-
20	Valencia Orangerene	C_15_H_24_	4630-07-3	-	1.42	-
21	Bicyclogermacrene	C_15_H_24_	24703-35-3	-	2.66	-
22	*α*-Himachalene	C_15_H_24_	3853-83-6	-	1.62	-
23	*β*-Bisabolene	C_15_H_24_	495-61-4	0.08	19.39	-
24	*β*-Curcumene	C_15_H_24_	28976-67-2	-	0.15	-
25	(*Z*)-Gamma-bisabolene	C_15_H_24_	13062-00-5	-	0.18	-
26	(+)-*δ*-Cadinene	C_15_H_24_	483-76-1	-	0.28	-
27	(*E*)-*α*-Bisabolene	C_15_H_24_	25532-79-0	-	0.3	-
28	Phenyl isothiocyanate	C_7_H_5_NS	103-72-0	-	0.22	-
29	Nonanal	C_9_H_18_O	124-19-6	0.07	0.23	-
30	(*Z*)-Citral	C_10_H_16_O	106-26-3	0.58	1.04	2.49
31	Citral	C_10_H_16_O	5392-40-5	0.05	1.9	-
32	(*E*)-Citral	C_10_H_16_O	141-27-5	0.62	-	2.97
33	Linalool	C_10_H_18_O	78-70-6	0.33	0.76	0.64
34	(-)-Terpinen-4-ol	C_10_H_18_O	20126-76-5	0.65	0.37	-
35	*α*-Terpineol	C_10_H_18_O	10482-56-1	0.72	2.05	0.89
36	Undecanal	C_11_H_22_O	112-44-7	-	0.17	-
37	4-Terpinyl acetate	C_12_H_20_O_2_	4821/4/9	-	0.18	-
38	dl-Citronellol acetate	C_12_H_22_O_2_	150-84-5	-	0.29	-
39	Nerylacetate	C_12_H_20_O_2_	141-12-8	0.25	6.76	-
40	Acetic acid geranyl ester	C_12_H_20_O_2_	105-87-3	0.09	3.67	-
41	1,1-Diethoxynonane	C_13_H_28_O_2_	54815-13-3	-	0.27	-
42	3,5,5-Trimethylnonyl Hexanoic acid	C_18_H_36_O_2_	1000406-06-0	-	0.13	-
Total detected components/each				22	39	9

“-” indicates that the content was less than 0.01 or not detected.

**Table 3:** Components and proportion of the three *lemon* peel extracts.

Items	LPEO	LPE	LPAO
Terpenes/%	96.64	81.96	93.01
Alcohols/%	1.70	3.18	1.53
Aldehydes/%	1.32	3.34	5.46
Esters/%	0.34	11.12	-
Other compounds/%	0.00	0.42	-
Total	100.00	100.00	100.00

**Table 4:** Content of total flavonoids and phenolic in three *lemon* peel extracts (*n* = 3).

No.	Total flavonoid/μg/ml	Total phenolic/μg/ml
LPEO	42.57 ± 0.05	32.06 ± 0.08
LPE	77.94 ± 0.03	61.24 ± 0.04
LPAO	62.72 ± 0.06	48.02 ± 0.07

**Table 5:** The peak odor and retention values of three *lemon* peel extracts.

NO.	0 s	60 s		100 s		140 s	
	Peak value/G_0_	Peak value/G	Aroma retention value/%	Peak value/G	Aroma retention value/%	Peak value/G	Aroma retention value/%
LPEO	43.4134	42.3630	97.58	41.7211	96.10	40.9111	94.24
LPE	20.3461	18.4422	90.64	17.0511	80.81	15.5736	76.54
LPAO	16.6896	16.3119	97.74	15.9618	95.64	15.3355	91.89
